# Mapping the Speech Code: Cortical Responses Linking the Perception and Production of Vowels

**DOI:** 10.3389/fnhum.2017.00161

**Published:** 2017-04-07

**Authors:** William L. Schuerman, Antje S. Meyer, James M. McQueen

**Affiliations:** ^1^Psychology of Language, Max Planck Institute for PsycholinguisticsNijmegen, Netherlands; ^2^Donders Institute for Brain, Cognition and Behaviour, Radboud UniversityNijmegen, Netherlands

**Keywords:** altered auditory feedback, categorical perception, EEG/ERP, sensorimotor integration, phonetics, speech production and perception

## Abstract

The acoustic realization of speech is constrained by the physical mechanisms by which it is produced. Yet for speech perception, the degree to which listeners utilize experience derived from speech production has long been debated. In the present study, we examined how sensorimotor adaptation during production may affect perception, and how this relationship may be reflected in early vs. late electrophysiological responses. Participants first performed a baseline speech production task, followed by a vowel categorization task during which EEG responses were recorded. In a subsequent speech production task, half the participants received shifted auditory feedback, leading most to alter their articulations. This was followed by a second, post-training vowel categorization task. We compared changes in vowel production to both behavioral and electrophysiological changes in vowel perception. No differences in phonetic categorization were observed between groups receiving altered or unaltered feedback. However, exploratory analyses revealed correlations between vocal motor behavior and phonetic categorization. EEG analyses revealed correlations between vocal motor behavior and cortical responses in both early and late time windows. These results suggest that participants' recent production behavior influenced subsequent vowel perception. We suggest that the change in perception can be best characterized as a mapping of acoustics onto articulation.

## Introduction

Learning to produce speech requires mapping acoustics onto articulation (Guenther, 1994; Kuhl, 2004). Sensory-to-motor mappings may be continuously updated during adulthood based on input from the environment (e.g., Sancier and Fowler, 1997) and sensorimotor experience (Brainard and Doupe, 2000; Tschida and Mooney, 2012). While the role of sensorimotor experience for maintaining production abilities is uncontroversial, the role of sensorimotor experience during speech perception has been highly contested (Hickok, 2009; Hickok et al., 2009; Wilson, 2009). More recently, the focus has shifted from investigating whether production systems are involved in perception to “unpacking” how production systems and production experience influence perception (e.g., Stasenko et al., 2013; Skipper et al., 2017). While sensory-to-motor mappings appear to be critical for developing speech production abilities, it is unclear to what extent perception may involve mapping acoustics onto articulation. Evidence suggests that more accurate speech perception correlates with more distinct articulation (Perkell et al., 2004a,b), pointing towards a close link between perception and production abilities. Yet it is not clear how changes in one system (e.g., perception) lead to changes in the other (e.g., production). In the present study, we examined cortical and behavioral responses during a vowel categorization task prior to and following sensorimotor training in order to investigate how sensorimotor experience affects the neural processing of speech sounds.

Phonetic categories that can differ by a single acoustic value, such as voice-onset-time (VOT), are divided by a perceptual boundary (Liberman et al., 1957), marking the point at which sound acoustics stop corresponding to one category and begin to correspond to the other. The location of this perceptual boundary along a continuum between two sound categories can be shifted as a result of experience, a phenomenon known as phonetic recalibration (Samuel and Kraljic, 2009). For example, by inserting an ambiguous fricative sound between [f] and [s] into a context in which hearing the sound as [s] would create a real word and [f] would not (e.g., pass vs. paff), listeners can be biased to perceive the sound as [s]. After repeated exposure to these biasing contexts, listeners are more likely to categorize the ambiguous sound as [s] in subsequent phonetic categorization tasks (Norris et al., 2003). Thus, experience that biases how acoustic values are categorized can lead to shifts in the perceptual boundary between two phonetic categories.

Recent experiments have found that sensorimotor adaptation can also lead to shifts in the perceptual boundary between two phonetic categories (Shiller et al., 2009; Lametti et al., 2014). Frequency alteration devices (e.g., Houde and Jordan, 1998, 2002) enable an experimenter to introduce a mismatch between a speaker's articulation and the acoustics of the resulting sound. A speaker may attempt to compensate for the shift by articulating in the opposite direction, though the degree of compensation is usually not sufficient to completely counteract the frequency shift (MacDonald et al., 2011; Katseff et al., 2012). After continued exposure to shifted feedback, the compensatory response may stabilize such that when producing a target sound, the speaker continues to utilize a newly learned articulation even when the altered feedback is masked or removed (Purcell and Munhall, 2006). At this point of stabilization, the speaker is considered to have “adapted” to the new sensorimotor mapping. Shiller et al. (2009) found that when participants' [s] productions were shifted down (towards values for [ʃ]), participants compensated by increasing the frequency of the fricative. Compared to baseline, this change in production behavior led participants to categorize more stimuli as [s] following training. In contrast, control participants who received unaltered feedback tended to categorize *fewer* stimuli as [s] after training.

In a related study, Lametti et al. (2014) found that changes in vowel articulation due to sensorimotor adaptation led to specific changes in phonetic categorization. Participants were first tested on their perception of a phonetic continuum between “head” and “hid” (Exp. 1) or “head” and “had” (Exp. 2). Then, during production training, participants produced the word ‘head’, while F1 was either increased (to sound more like “had”) or decreased (to sound more like “hid”). Following sensorimotor adaptation, participants who *articulated* into the test region (e.g., producing “head” more like ‘hid’, then tested on a head-to-hid continuum) were found to show a decrease in the proportion of stimuli labeled as ‘head’. No changes in categorization were found for the opposite shift or control participants. While neither study found significant correlations between the magnitude of adaptation and the magnitude of change in perceptual function, such studies demonstrate that sensorimotor adaptation can lead to changes in phonetic categorization.

However, it is unknown whether the effects observed in these experiments stem from changes to early stages of speech sound processing, e.g., acoustic encoding or feature extraction, or later stages involving perceptual decision making (Norris et al., 2000). This distinction is crucial in order to relate these effects to speech perception under typical listening conditions, as changes to late stage processes may only affect performance on specific laboratory tasks (Hickok and Poeppel, 2000, 2007). Furthermore, examining how a listener's sensorimotor experiences alter the processing of sounds may elucidate the role of sensorimotor integration in speech perception (Hickok et al., 2011).

We consider two primary time-windows at which sensorimotor experience may affect speech sound processing. The first is an early window around 100ms after stimulus onset, corresponding to the N1/M1 electrophysiological components. The N1/M1 has been described as an “exogenous” response (Picton, 2013), reflecting the acoustic properties of the stimulus. Accordingly, repeated presentation of a speech stimulus leads to suppression of this component, while actively imagining the same stimulus prior to presentation does not (Tian and Poeppel, 2013).

The identity of a perceived vowel can be predicted based on early tonotopic activity in primary auditory cortex (Chang et al., 2010) that encodes the acoustic features relevant for distinguishing vowels from each other. Many vowels can be described as a combination of the first two resonating frequencies, or *formants*, of the vocal tract (F1 and F2). The values of these formants correspond to the height of the jaw and tongue body (F1) and the anteriority/posteriority of the tongue body (F2) (Fant, 1960). Vowels varying along these two dimensions elicit distinct cortical responses as early as 100ms after stimulus onset (Obleser et al., 2003a,b, 2004; Shestakova et al., 2004). Based on these data, early auditory activity around 100ms may reflect acoustic feature extraction (Tavabi et al., 2007) or pre-lexical abstraction (Obleser and Eisner, 2009).

While long term changes in the amplitude of the N1/M1 auditory component have been found after musical training (Pantev et al., 1998), the amplitude of activity in this time-region can also be modulated by attention (Poeppel et al., 1997). Hickok et al. (2011) have speculated that forward predictions based on prior sensorimotor experience direct attention to relevant acoustic features of an expected sound, possibly modulating the gain and response selectivity of neurons tuned to those features. If sensorimotor experience can affect how features are extracted or encoded, e.g., by altering the degree of vowel “height” encoded by a particular F1 value or the degree of vowel “frontness” encoded by F2, then this ought to be reflected by changes in N1 amplitude.

The second time window we consider is centered around 200 ms (P2/M2) and has been associated with perceptual decision making (Mostert et al., 2015) as well as phonological processing (Tian and Poeppel, 2013). While it may be possible to decode vowel identity from distributed activity in early processing stages (Chang et al., 2010), in a phonetic categorization task this neuronal activity must ultimately be linked to a linguistic representation in order to produce a behavioral response (Poeppel et al., 2008). Phonetic categorization involves mapping a stimulus exemplar drawn from a continuous acoustic distribution onto a discrete category. Typical response patterns from experiments involving binary decisions generate sigmoidal response curves that mark the boundary between the two phonetic categories (as, for example, in the present experiment; see **Figure 2**). Due the transformation from a continuous acoustic space to a binary response space, behavior in sensory decision tasks may not directly reflect sensory encoding but subsequent decision processes *acting upon* sensory representations (Mostert et al., 2015). Accordingly, behavioral responses in a phonetic categorization task have been found to correlate with variations in the amplitude of the event-related P2 component (but not the earlier N1; Bidelman et al., 2013).

Auditory training with speech stimuli, in which participants respond to training stimuli with non-vocal responses (e.g., button presses), has been found to modulate P2 amplitude. The effects of auditory training on cortical responses has been investigated extensively with regard to the perceptual learning of VOT contrasts (Tremblay et al., 1997, 2001). This series of auditory training and auditory exposure studies revealed that P2 amplitude increases in response to repeated exposure to a training continuum, regardless of change in perceptual performance (Tremblay et al., 2009, 2010; Sheehan et al., 2005; Alain et al., 2010). Researchers have consequently suggested that increases in P2 amplitude are general biomarkers of auditory learning, possibly representing a first-stage process involving auditory object familiarization and representation (Tremblay et al., 2014). However, Tremblay et al. (2014) specifically ascribed the increased P2 amplitude to the context of learning a *novel* (i.e., unknown, non-native) phonetic contrast. Such contrasts may be represented as distinct auditory objects (Ross et al., 2013). Therefore, it is unclear from these studies whether changes to *existing* phonetic contrasts also involves modulation of this component.

Electrophysiological experiments on native speech categories suggest that phonetic recalibration results in changes to later perceptual decision components. Utilizing a mismatch negativity paradigm, van Linden et al. (2007) exposed participants to an ambiguous consonant, midway between [t] and [p]. This ambiguous stimulus was utilized as the standard, and compared with a deviant which was an unambiguous [t]. By altering the lexical context in which this ambiguous stimulus was embedded, listeners were biased to hear the ambiguous consonant as either a [p] or a [t]. In a previous behavioral study, this manipulation was found elicit a shift in the phonetic categorization boundary between [p] and [t] (Van Linden and Vroomen, 2007). A significant mismatch negativity was elicited when the standard was heard as [p] but not when it has been heard as [t], suggesting that biasing the listeners to categorize the ambiguous stimulus as a member of another phonetic category resulted in greater perceptual distance between standard and deviant (van Linden et al., 2007). Furthermore, the peak MMN response was found at 215ms after segment onset, approximately the same time-region implicated in auditory perceptual learning (Tremblay et al., 2014). Regarding the current study, if the processes involved in phonetic recalibration are similar for both sensory (Samuel and Kraljic, 2009) and sensorimotor (Lametti et al., 2014) training, then we may also expect sensorimotor training to elicit changes in cortical amplitude in this late time window.

Ito et al. (2016) examined the effects of sensorimotor adaptation on auditory potentials recorded in response to a single unambiguous [ɛ] vowel, which was presented before and after speech motor training. This motor training involved shifting auditory feedback during production of the word “head” such that the participants heard themselves producing a vowel more like the one in “hid” (by decreasing F1). In order to counteract this shift in feedback, participants would therefore have to produce a vowel more like that in “had” (by increasing F1). Participants were divided into three groups of equal size based on whether they had produced consistent compensatory motor behavior opposing the feedback shift (adapted), had failed to compensate for the shifted feedback (non-adapted), or had instead received unaltered feedback (control). Only in the adapted group did the authors find a significant change in the amplitude of the P2 component. In contrast to the increases in P2 amplitude found in perceptual learning studies (Tremblay et al., 2010, 2014), adapters exhibited a *decrease* in P2 amplitude over right frontal electrodes. While the interpretation of the decreased P2 amplitude was not entirely clear, the timing of the effect was in line with previous research on phonetic recalibration and perceptual learning (van Linden et al., 2007; Tremblay et al., 2014).

To summarize, previous research has found that sensorimotor adaptation to altered auditory feedback during speech production alters phonetic categorization (Lametti et al., 2014). The latency of sensorimotor adaptation effects on cortical responses (Ito et al., 2016) suggests that sensorimotor adaptation modulates activity in processing stages associated with phonetic categorization (Bidelman et al., 2013) rather than acoustic encoding (Obleser and Eisner, 2009).

**Figure 1:**
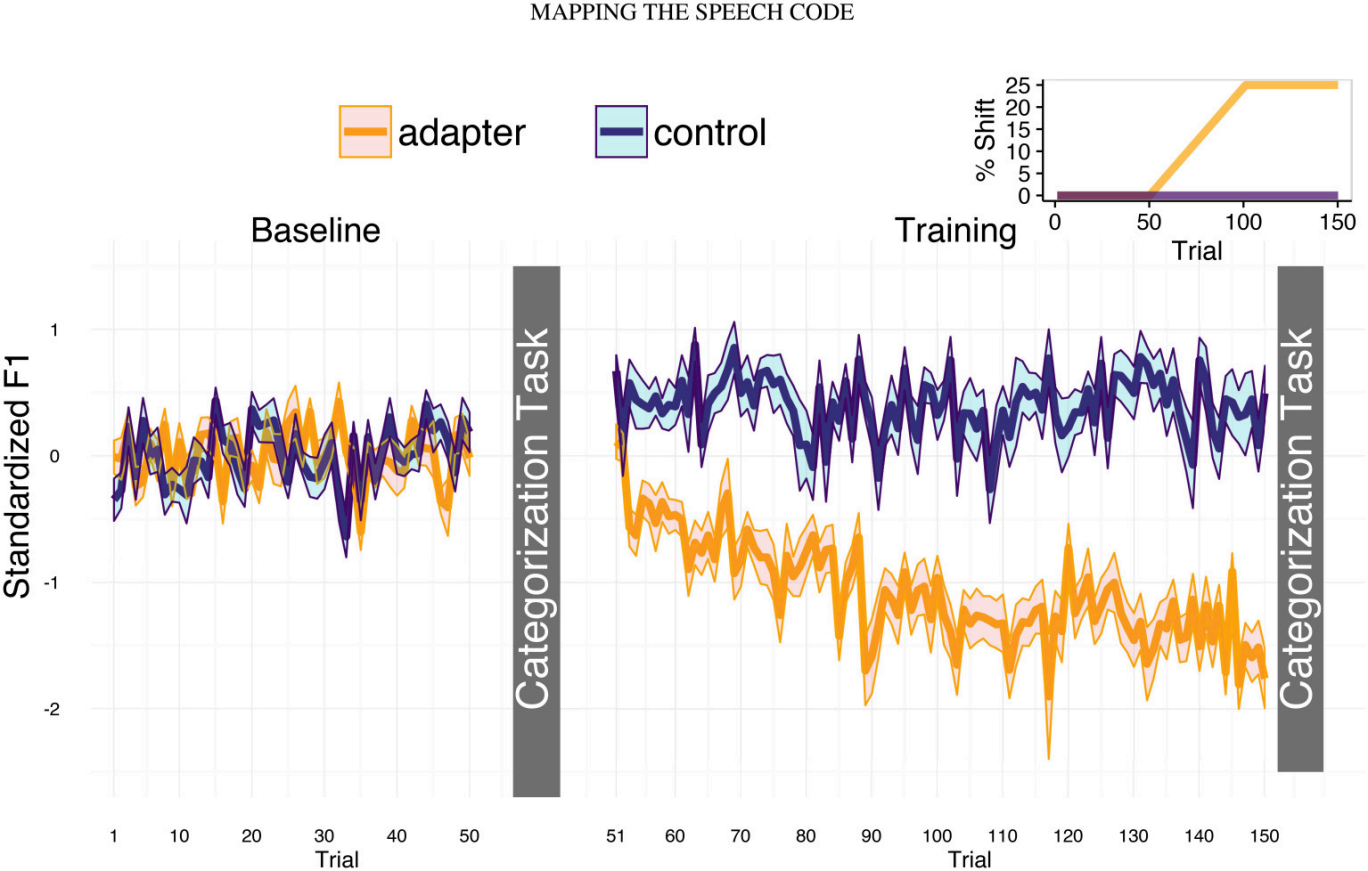
**Experimental design and production data**. In the two speaking task (baseline and training), participants repeatedly produced the Dutch word “pet” (cap), which contains the front-mid vowel [ɛ]. Phonetic categorization tasks took place immediately after the end of the baseline (trial 50) and training sessions (trial 150). For both groups, feedback was unaltered during the baseline session (left panel). For the altered feedback group, the value of F1 in the auditory feedback increased linearly between trials 51 and 70 to a maximum of 25% greater than each trial's original value (inset, top right). Thus, participants heard themselves producing a more [æ]-like vowel, which led to compensatory decreases in F1 (yellow line). Feedback was unaltered for controls (purple line).

The present study sought to build upon these results in order to further explore how speech perception may reflect sensorimotor experience. We compared behavioral and cortical responses during a phonetic categorization task prior to and following sensorimotor training of speech production. Dutch participants were recorded producing the Dutch word “pet” (“cap”) containing the front mid-vowel [ɛ], and then performed a phonetic categorization task during which EEG was recorded. For the categorization task, we parametrically varied values of F1 to create a five-step continuum between [ɛ] and [ɪ]. In the subsequent speech training session, half of the participants were exposed to altered auditory feedback (the AF group) while the other half received unaltered feedback (the UF group). For the AF group, the value of F1 was increased, which caused participants to hear themselves producing a vowel more like [æ]. Compensating for this shifted feedback required articulating into a motor space that would normally produce a sound between [ɛ] and [ɪ], which in previous experiments had been found to lead to phonetic recalibration (Lametti et al., 2014). This training session was followed by another phonetic categorization task (Figure 1). The design enabled us to examine how changes in phonetic recalibration were related to changes in speech motor behavior, and how changes in speech motor behavior and phonetic categorization were reflected in electrophysiological responses.

In Lametti et al. (2014), articulating [ɛ] as a more [ɪ]-like vowel led to increases in the proportion of stimuli categorized as [ɪ]. We therefore expected that adapters (AF group) would categorize more stimuli as [ɪ] after sensorimotor adaptation, while controls (UF group) would not. However, while we observed significant adaptation in response to the altered feedback, we found no significant differences between groups in changes in phonetic categorization after sensorimotor training.

We therefore conducted systematic exploratory analyses to examine to what extent individual differences in the production of the training vowel ([ɛ]) corresponded to behavior in the phonetic categorization tasks and electrophysiological data (cf. Bradlow et al., 1996). Though no significant correlations between perceptual and motoric behavior were found in Lametti et al. (2014), based on their group level results we expected that decreases in F1 (articulating an [ɛ] as a more [ɪ]-like vowel) should correlate with an increase in the proportion of stimuli categorized as [ɪ], though possibly only for participants who received altered auditory feedback.

Regarding the electrophysiological data, if auditory-motor remappings lead to changes in vowel encoding, then this should modulate the amplitude of the early N1 component. If, auditory-motor remappings cause changes in perceptual decision making, then this ought to modulate the later P2. With respect to the direction of this modulation, prior evidence leads to conflicting predictions. While exposure to a phonetic continuum leads to increases in P2 amplitude (e.g., Tremblay et al., 2014), sensorimotor adaptation has been found to lead to decreases in P2 amplitude (Ito et al., 2016). However, the fact that the sensorimotor adaptation used only a single test vowel, rather than a continuum, may have led to this discrepancy in the results. If exposure to a phonetic continnum leads to increases in P2 amplitude, while sensorimotor adaptation leads to decreases in P2 amplitude, we may expect these effects to cancel out for adapters (or go in opposite directions for specific stimuli), while controls should only exhibit increases in P2 amplitude.

Rather than deriving our expectations about changes to neural components solely from the type of feedback participants receive, alternatively, we can generate predictions about the direction of neural component change based on what would be expected from a sensory-to-motor mapping. Bidelman et al. (2013) found that when an ambiguous vowel between [a] and [u] was classified as [u] (closed jaw position/low F1), P2 amplitude was lower than when the same vowel was perceived as [a] (open jaw position/high F1). Thus, there is a positive relationship between F1/openness and P2 amplitude. If the vowel [ɛ] in “pet” comes to be associated with a more closed or open jaw position due to changes in production (regardless of feedback), then a sensory-to-motor mapping account would predict that P2 amplitude should increase or decrease correspondingly. In Ito et al. (2016), adapters that produced [ɛ] with a more open jaw position (higher F1) exhibited greater reduction in P2 amplitude. In terms of a sensory-to-motor mapping, this suggests that after training, the test vowel corresponded to a lower F1/more closed jaw position. If adapters in the current experiment produce [ɛ] with a more closed jaw position (lower F1), we would expect to observe the opposite effect, that after training the test vowel would be perceived as corresponding to a higher F1/more open jaw position, and P2 amplitude should increase accordingly.

## Materials and methods

### Participants

A total of 48 native Dutch speakers took part in the study. All reported normal hearing and vision. Previous experiments had found that some participants do not exhibit changes in articulatory behavior in response to altered auditory feedback (MacDonald et al., 2011). Twenty-eight participants were assigned to the altered feedback condition. Of these, twenty produced significant articulatory responses opposing the direction of the shifted feedback (see Results: speech adaptation; average age = 21.3, range = 18–28, four men). We then recruited an additional twenty participants to serve as a control group (unaltered feedback; average age = 22, range = 19–30, five men).

Ethical approval for this study was obtained from the Ethics Committee of the Social Sciences Faculty of Radboud University. Participants were informed that their participation was voluntary and that they were free to withdraw from the study at any time without any negative repercussions and without needing to specify a reason for withdrawal. Written consent was obtained from each participant and all were reimbursed for their participation.

### Procedure

After cap fitting, participants were led to a recording booth for the production baseline. Participants were then led back to the EEG recording booth for the perception pre-test. They then returned to the recording booth for production training, and then once again returned to the EEG booth and performed the listening post-test (which was identical in design to the pre-test). In order to ensure that listening effects in the post-training phase were based solely on the feedback received during training and not from vicarious inter-session speech, all participants were instructed to not communicate verbally (unless absolutely needed) between the training phase and the listening post-test, while the researcher also refrained from any verbal communication.

### Speaking—baseline and training

Production tasks took place in a sound-attenuated booth. Speech recording and auditory feedback transmission was carried out using Audapter (Cai et al., 2008; Tourville et al., 2013), a feedback manipulation program implemented in Matlab (Mathworks, 2012). Participants were seated in a chair approximately 5–10 cm away from a pop-filter shielded microphone (Sennheiser ME 64) and fitted with sound isolating headphones (Sennheiser 280). The volume of the headphones was calibrated individually such that participants reported only being able to hear their voice through the headphones, masking their actual productions. Yet we also ensured that the level of the volume caused no physical discomfort. If participants began to whisper or speak too softly, an automated warning message appeared on screen asking them to increase their speaking volume. Broadband noise (60 dB) was added to the auditory feedback signal in order to further mask bone-conducted sound (Békésy, 1949).

Speech tokens were elicited by visual presentation of the orthographic form of the target word. In the baseline phase, participants first produced four instances of the words “pit” (pit) and “pet” (cap/hat; containing the vowels [ɪ] and [ɛ], respectively) in random order. They then produced 50 repetitions of word “pet”. Averaged F1 measurements in these 50 trials constituted each participant's production baseline.

In the training phase, participants repeated the Dutch word “pet” (“cap”) 100 times. For participants in the control group, there were no modifications to the spectral parameters of the participants' utterances (Unaltered Feedback; UF). However, the auditory signal was transmitted through the same speech modulation software in order to generate the same delay and masking noise experienced by the altered feedback group. For the altered feedback (AF) group, we implemented a slightly modified version of the paradigm utilized in Lametti et al. (2014). For this group, the frequency of the first formant was shifted upwards by 25%. If a participant produced the word “pet” ([pɛt]) with normal articulation, a 25% increase in the first formant would result in the participant hearing themself producing something sounding like the English word “pat” ([pæt]). The intensity of the feedback shift increased linearly from between trials 1 through 30 and was held constant for the remainder of the training session.

### Listening—EEG data acquisition and preprocessing

EEG data acquisition took place during the two listening tasks following the production baseline and training sessions. Participants were seated comfortably in front of a computer screen and a button-box. Auditory stimuli were emitted from two speakers flanking the computer screen. Stimulus delivery and response monitoring was controlled using Presentation (Version 0.70, www.neurobs.com). Each trial began with a blank screen. Auditory stimulus presentation began after a random waiting interval between 400 and 600ms (in increments of 20ms; Bidelman et al., 2013). Participants attempted to respond as quickly as possible by pressing one of two buttons corresponding to two phonetic categories: “korte e” (“short e”, [ɛ]) and “korte i” (“short i”, [ɪ]). All participants responded using the index and middle fingers of their dominant hand.

Auditory stimuli comprised a five-step Klatt-synthesized vowel continuum between clear [ɛ] and clear [ɪ]. Pitch and formant values were based on average values for a female speaker of Dutch (Schuerman et al., 2015). Stimuli were presented 100 times each. Sessions thus consisted of a total of 500 trials, with self-paced rest periods after every 100 trials. Presentation was randomized such 20 presentations of each stimulus occurred in each block, and every possible two-way combination of stimuli (e.g., step 1 followed by step 1, step 1 followed by step 2…) occurred an equal number of times.

Continuous electroencephalograms (EEGs) were recorded using a 32 electrode Acti-Cap system, with reference electrodes placed on both mastoids (**Figure 4A**). Two additional electrodes were placed above and below the left eye to measure blinks and eye-movements. A common ground electrode was placed along the midline (AFz), with reference electrode on the left mastoid. EEG data was later re-referenced to paired electrodes on both left and right mastoids.

EEGs were sampled at 20 kHz and online filtered between 0.05 and 3500 Hz. Artifact rejection and averaging was conducted in Matlab (Mathworks, 2012) using the Fieldtrip toolbox (Oostenveld et al., 2011). Event-related potentials (ERPs) were baselined with respect to -100ms prior to stimulus onset and windowed from -100ms pre-stimulus onset to 600ms post-stimulus onset. Prior to artifact rejection, a band-stop filter with a 50Hz center frequency and 1Hz bandwidth was applied to eliminate machine noise. Semi-automated artifact rejection was employed, in which likely artifacts were marked. All trials containing artifacts were removed after visual inspection. This resulted in a total of 4227 rejected trials (22.1% of data; average 52.2 trials per participant). For each participant, independent component analysis was used to identify and remove eye-blink and heartbeat related components. Prior to averaging, the remaining ERPs were low-pass filtered at 30Hz to isolate cortical responses.

### Acoustic analysis

Recordings from the production task were analyzed with Praat (Boersma and Weenink, 2016). The vocalic section of each recording was automatically located, and vowel measurements taken from the midpoint. The values of the first and second resonant frequencies of the vocal tract (formants; F1 and F2) were calculated using 10ms overlapping windows, and tracked using 12 LPC coefficients. Formant values exceeding five standard deviations above or below each participants average F1 value were excluded as these values were likely the result of tracking errors. Formant values in Hertz were converted to Mels, a logarithmic frequency scale based on the properties of human hearing, using the formula 2595 * (*log*(1 + (*F*_*Hz*/700))).

In order to compare participants having differing vocal tracts with respect to changes in production, formant values were standardized relative to each participant's average values during the baseline speaking task, according to the following equation (where F refers to F1 or F2):

(1) $$\begin{array}{r} F_{standardized}=\left(F-mean\left(F_{baseline}\right)\right)/sd{\left(F\right)}_{baseline} \end{array}$$

### Event related potential analysis

Event-related potentials were analyzed using nonparametric cluster-based permutation analysis (Maris and Oostenveld, 2007), which is well suited to exploratory comparisons between two groups. This method utilizes an algorithm based on the assumption that ERP effects are clustered over both space and time in order to address the family-wise error rate arising from multiple comparisons. For each sample, the candidate contrasts are compared at each channel and each time point using *t*-tests. Next, all samples with t-values larger than a specified threshold are selected, while all samples failing to meet this threshold are discarded. Selected samples are then clustered on the basis of temporal and spatial distance, and t-values are summed over these clusters. In order to generate the null distribution against which this test statistic is compared, trials from the two conditions are randomly partitioned into two subsets and summed t-values are calculated from these randomly generated clusters. In this experiment, the number of permutations per contrast was set at 10000. The test statistics of these randomly generated partitions are compared to the test statistic of the experimentally observed data, and the proportion of partitions greater than the observed partition constitutes the significance level of the cluster (i.e., its *p-value*). In the current experiment, within- and between-group contrasts were tested using a two-tailed alpha level of 0.025. Tests for significant clusters in the interaction between session and group utilized an alpha of 0.05.

## Results

All statistical analyses were implemented in R (R Core Team, 2013). ANOVAs were implemented using the *ez* package (Lawrence, 2011). Bayesian ANOVAs were calculated the package *BayesFactor* (Morey et al., 2015) and Bayesian correlations were conducted using the package *BayesMed* (Nuijten et al., 2015). In cases where the distribution of the data did not allow for parametric testing, the appropriate non-parametric version was utilized.

### Speech production training

For participants in the altered feedback group, adaptation was assessed utilizing one-tailed independent sample *t*-tests between the baseline (50 trials) and the last 50 trials of the training phase (hold phase) for each individual. Out of 28 participants, 20 “adapters” exhibited significant compensatory decreases in F1, opposing the shift in auditory feedback. The remaining eight participants were excluded from the subsequent analysis. We first tested for potential phonetic differences between groups during the baseline and training sessions. While the experimental manipulation targeted F1, speakers have been found to alter their production of unshifted formants in response to altered auditory feedback (MacDonald et al., 2011). Therefore, in addition to F1, we also analyzed F2.

An ANOVA on produced F1, with type as a between-subjects factor and session as a within-subjects factor, revealed a significant main effect of session (*F*_(38)_ = 5.676, *p* = 0.022, *ges* = 0.011), as well as a significant interaction between session and type (*F*_(38)_ = 41.448, *p* < 0.001, *ges* = 0.077). Subsequent two-sample *t*-tests (with Levene tests for equal variance) indicated that F1 differed significantly between groups in the training session (*t*_(38)_ = 3.618, *p* < 0.001, *BF*_10_ = 35.26), but not in the baseline session (*t*_(38)_ = 0.021, *p* = 0.98, *BF*_10_ = 0.31). Thus, groups did not differ on F1 during baseline but did differ significantly during the training session.

For F2, only a significant interaction between session and type was found (*F*_(38)_ = 8.523, *p* = 0.006). However, the effect size was extremely small (*ges* = 0.003), and post-hoc tests indicated no significant differences in F2 between groups in either the baseline (*p* = 0.85, *BF*_10_ = 0.31) or training sessions (*p* = 0.396, *BF*_10_ = 0.41).

In the hold phase of the training session, controls' exhibited an average increase in F1 of 43.69_*mel*_ relative to baseline, while adapters exhibited an average decrease of −94.54_*mel*_. In order to assess changes in formant production between sessions, we standardized formant values with respect to the mean and standard deviation of each participant's baseline. Figure 1 displays the average values for standardized F1.

Two-sample *t*-tests confirmed that average standardized F1 differed significantly between adapters and controls (*t*_(38)_ = −5.859, *p* < 0.001, *BF*_10_ = 13941.8). A Wilcox test indicated that standardized F1 was significantly below baseline for participants in the AF group (*p* < 0.001). At first, standardized F1 in the control group did not appear to differ significantly from baseline. However, after removing one possible outlier (*z* = −2.93), standardized F1 was found to be significantly greater than baseline in the control group (*t*_(18)_ = 3.680, *p* = 0.002). Thus, these groups exhibited divergent F1 productions during the speech production tasks.

Standardized F2 differed significantly between adapters and controls as well (*t*_(38)_ = 2.4567, *p* = 0.019, *BF*_10_ = 3.09). Standardized F2 differed significantly from baseline for controls (*t*_(18)_ = −2.28, *p* = 0.034) but not adapters (*p* = 0.17).

Two-tailed *t*-tests for each participant in the control group revealed that nine participants exhibited statistically significant increases in F1 compared to baseline, while two exhibited significance decreases. Similarly, eight controls and five adapters exhibited significant decreases in standardized F2, while three controls and eight adapters exhibited significant increases in standardized F2. Therefore, both within and across groups, participants exhibited variable patterns of vocal motor behavior, not only for the altered formant but in other formants as well.

### Phonetic categorization

**Figure 2:**
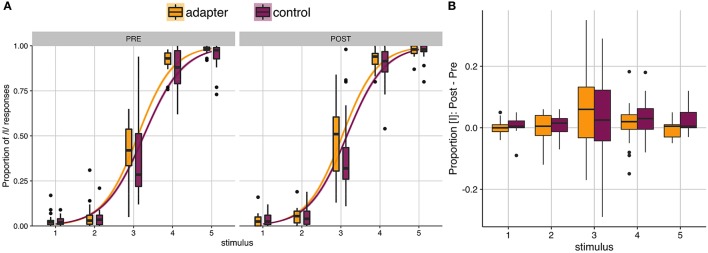
**Phonetic categorization**. **(A)** Results of phonetic categorization tasks before and after speech production training. After baseline (left panel) and training (right panel) speaking sessions, participants categorized vocalic stimuli as either [ɛ] or [ɪ]. The proportion of [ɪ] responses is indicated on the y-axis, as a function of stimulus step (x-axis). Yellow (adapters) and purple (controls) lines indicate the slope of a logistic function fit to individual responses. Box plots indicate distribution of results across participants. No significant differences were found between groups in either session. **(B)** Box plots of average change in the proportion of stimuli categorized as [ɪ] by stimulus. Most participants tended to categorize all stimulus steps more often as [ɪ] in the post-test session. Outliers indicated by filled circles.

Prior to speech production training, control participants were found to have categorized slightly fewer stimuli as [ɪ] than adapters (2A, left panel). Following training, both groups categorized more stimuli as [ɪ] (Figure 2A, right panel). The average proportion of stimuli categorized as [ɪ] was analyzed using repeated-measures ANOVA (with Greenhouse-Geisser corrections), with group (adapters/controls) as a between-subjects variable and stimulus (5 levels), session (two levels), and block (5 levels) as within-subjects variables. Significant main effects were found for stimulus (*F*_(4)_ = 883.29, *p* < 0.001, *ges* = 0.898) and block (*F*_(4)_ = 3.741, *p* = 0.01, *ges* = 0.007). The main effect of block, with no interaction, indicated that for both groups, the proportion of [ɪ] responses tended to increase over both sessions (Table 1). A significant interaction was also found between stimulus and session (*F*_(4)_ = 3.379, *p* = 0.044, *ges* = 0.004), though as this effect did not pertain to our hypothesis it was excluded from further investigation. Unlike in Lametti et al. (2014), there was no significant effect of group and no interaction between group and session (all *ps* ≧ 0.116).

**Table 1:** **Average proportion of [ɪ] responses by block, session, and group**.

**Session**	**PRE**					**POST**				
**Block**	**1**	**2**	**3**	**4**	**5**	**1**	**2**	**3**	**4**	**5**
Adapters	0.46	0.47	0.5	0.48	0.49	0.48	0.50	0.52	0.49	0.48
Controls	0.43	0.43	0.44	0.48	0.46	0.46	0.46	0.47	0.49	0.47

While between-group differences failed to reach significance, participants in both groups exhibited a large amount of variation in behavioral responses (Figures 2A–B). This variation between participants was the object of our exploratory analyses. We specified several potential relationships of interest between vocal motor behavior (quantified as F1 and F2) and phonetic categorization. These included: (1) Correlations between average F1 and F2 values in the baseline production task and average proportion of [ɪ]-responses in the subsequent perception task; (2) Correlations between average F1 and F2 values in the hold phase of the training session and average proportion of [ɪ]-responses in the subsequent perception task; (3) Correlations between standardized F1 and F2 (representing the change in formant values with respect to each participant's baseline) and the between session difference in average proportion of [ɪ]-responses. This totaled six correlations.

While none of these correlations was found to be significant after applying Holm-Bonferroni corrections for multiple testing (Holm, 1979), Bayesian analyses suggested some evidence of possible correlations between vocal motor behavior and perceptual responses. We therefore report the correlations with Bayes factors and the corresponding non-significant *p*-values.

**Figure 3:**
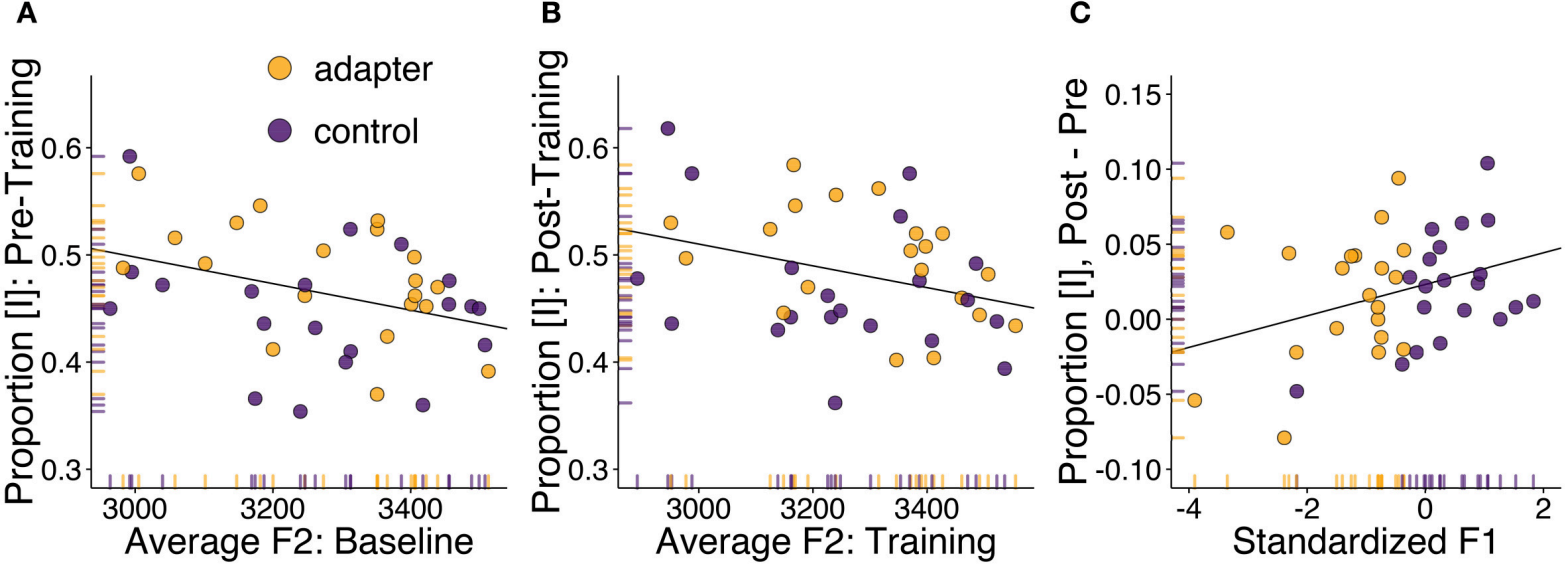
**Correlation analyses between speech motor behavior and phonetic identification**. Adapters are shown in yellow, controls in purple. **(A)** Correlation between F2 (in mels; averaged over all stimulus steps) for the word “pet” produced in the baseline session and the proportion of stimuli categorized as [ɪ] in the following phonetic categorization task. **(B)** Correlation between F2 in the speech training session and post-training phonetic categorization. **(C)** Correlation between standardized F1 (representing change in F1 in the speech training session compared to baseline) and the difference in phonetic categorization before and after speech training.

Due to the presence of significant correlations between F1 and F2 in both sessions, we conducted partial correlations controlling for the value of the other formant. Correlation tests between average [ɪ]-responses reported in the pre-training session and average F2 produced during baseline suggested a potential relationship (Figure 3A; *r* = −0.37, *p* = 0.022, *BF*_10_ = 2.42). The correlation between average F2 in the training session and the proportion of [ɪ] responses post-training did not reach significance (*r* = −0.27, *p* = 0.096, *BF*_10_ = 0.72). Our tests did not suggest, in either session, any relationship between average proportion of [ɪ] responses and F1.

A potential relationship was also found between standardized F1, indicating how F1 changed in the training session with respect to each participant's baseline, and between-session changes in the proportion of stimuli categorized as [ɪ] (Figure 3C; *r* = 0.34, *p* = 0.034, *BF*_10_ = 1.17). The direction of the correlation indicates that participants who produced vowels with a lower F1 during training (primarily adapters), tended to categorize fewer stimuli as [ɪ], while participants who produced vowels with a higher F1 tended to categorize more stimuli as [ɪ]. These results run contrary to those of Lametti et al. (2014), in which similar adaptation was found to correspond to a group-level increase in [ɪ]-responses.

Follow-up within-group exploratory tests suggested no group-specific correlations. No correlations with standardized F2 were found.

These analyses suggest that overall differences in phonetic categorization may have been related to differences in vocal motor behavior, though unexpectedly, the primary locus of these individual differences was found in variation in F2, not F1. Conversely, between-session changes in phonetic categorization were potentially reflected in standardized F1 but not standardized F2. This may have been due to the greater amount of variation in this formant's value between sessions compared to standardized F2. In both cases, these correlations suggest that the participants' perception of the phonetic continuum may have been influenced by their immediately preceding vocal motor behavior.

### Event related potentials

**Figure 4:**
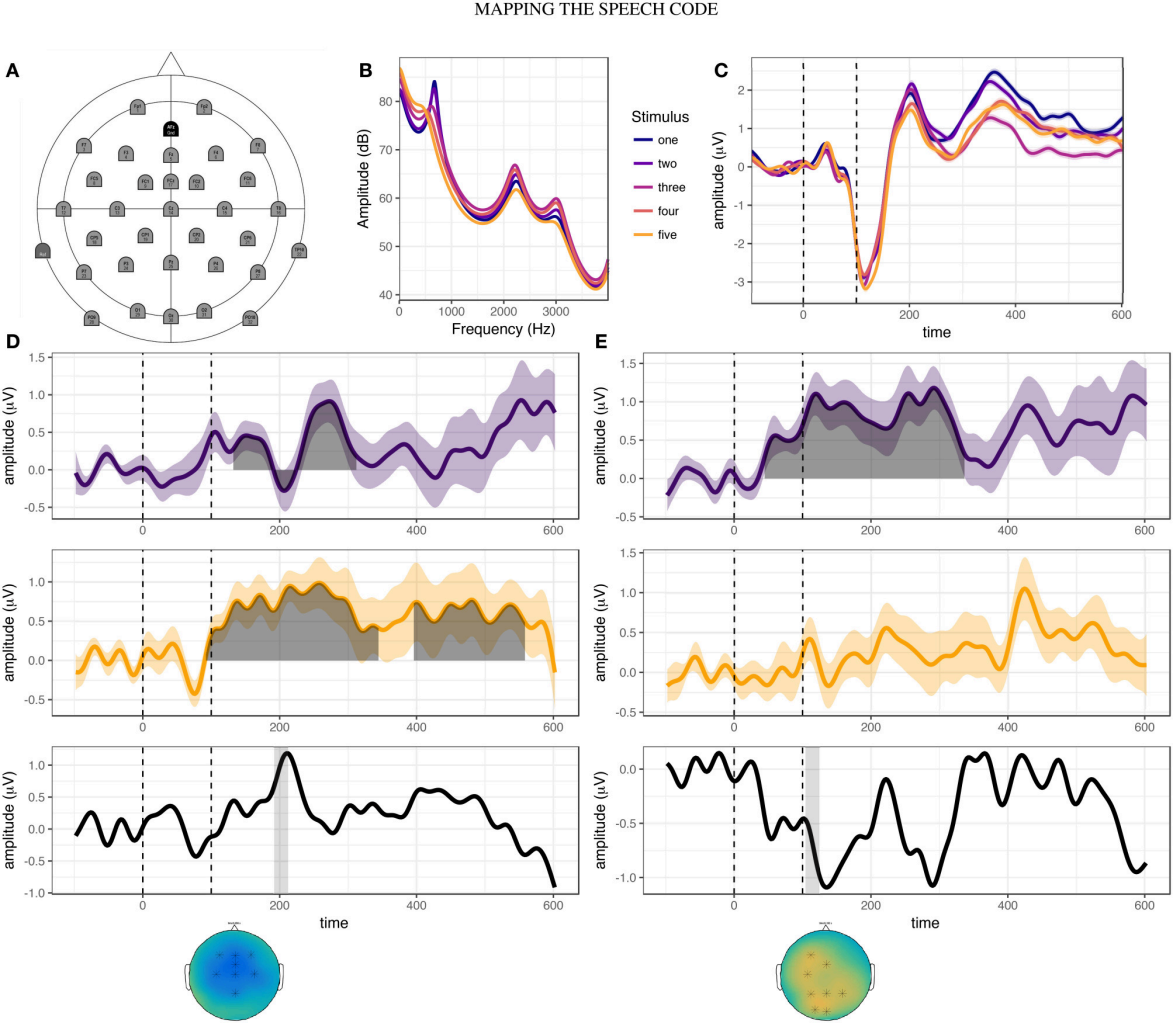
**Overview of EEG acquisition and results**. **(A)** Electrode layout for EEG acquisition. **(B)** Frequency-power spectrum of five stimulus steps ranging from [ɛ] (step one) to [ɪ] (step five). **(C)** Cortical responses to each stimulus step during the pre-training phonetic categorization task, averaged over all participants. **(D)** Results of cluster-based permutation analyses for stimulus step one. Between-session difference waves are shown for controls (purple line) and adapters (yellow line). Shaded region indicates time-window of significant cluster(s). Black line (bottom-panel) represents the subtraction of these two difference waves, revealing peak differences at approximately 210 ms after stimulus onset. Gray bar indicates 20ms time-window centered around the peak of the P2 event related component (192:212 ms). The distribution of cortical activity and electrodes found to be significant in the interaction cluster (marked by asterisk) are shown in the topographical plot below the bottom panel. **(E)** Results of cluster-based permutation analyses for stimulus step three, for controls and adapters. Gray bar indicates 20 ms time-window centered around the peak of the N1 event related component (104:124 ms). Topographical plots indicate the distribution of activity averaged over the time window, with significant electrodes indicated by asterisks.

Having observed evidence of potential relationships between the production and categorization tasks, we examined whether similar relationships may be found between behavior in the production tasks and electrophysiological responses. Auditory potentials to each stimulus step, averaged over all participants in the pre-test session, are shown in Figure 4C. In contrast to the results of Bidelman et al. (2013), stimulus steps two and three elicited greater P2 amplitudes than stimulus one. This suggests that for the [ɛ] - [ɪ] continuum utilized in this study (Figure 4B), P2 amplitude may not directly reflect distinctions in vowel height.

In the behavioral results, we found evidence suggesting that standardized F1 (representing the change in F1 between sessions) and changes in the proportion of stimuli categorized as [ɪ] may have been related (Figure 3C). We also found a potential correlation between the F2 produced by the participants and phonetic categorization in the baseline session (Figure 3A). These correlations suggest that the perceptual processing of the vocalic stimuli may have been related to how the participants produced the target vowel during baseline and training. If true, then this predicts that production variables may be reflected in cortical responses recorded during vowel categorization.

We first examined whether standardized F1 was related to either average or stimulus-specific cortical amplitude. In order to do so, it was necessary to identify specific electrodes and time windows over which to average cortical activity. We began by conducting an omnibus ANOVA, testing for main effects of group or interactions between group and session over specific electrodes. No significant effects of group or interactions with group were found.

We therefore decided to investigate stimulus-specific differences in cortical responses using cluster-based permutation analyses (Maris and Oostenveld, 2007). For between-groups contrasts, we found no significant clusters in the pre-training or post-training sessions for any stimulus step. This mirrors the results of the phonetic categorization task, in which no group level differences were found prior to or following speech training. However, within both the adapter and control groups, significant differences were found for between-session contrasts. The results of these within-group analyses are summarized in Table 2. For adapters, cortical responses to the endpoint stimuli (one and five) differed most between sessions, while for controls the greatest differences were observed for the most ambiguous stimulus steps (two, three, and four).

**Table 2:** **Results of cluster-based permutation analyses on within-group, between-session effects**.

**Stimulus**	**Controls**	**Adapters**
One	[132 – 312] *p* = 0.022	[92 – 344] *p* = 0.004 [396 – 558] *p* = 0.042
Two	[100 – 202] *p* = 0.03, [230 – 336] *p* = 0.023	[360 – 586] *p* = 0.017
Three	[046 – 336] *p* = 0.002	—
Four	[110 – 402] *p* = 0.008	[146 – 358] *p* = 0.016 [476 – 600] *p* = 0.042
Five	[212 – 380] *p* = 0.012	[090 – 338] *p* = 0.0041 [336 – 600] *p* = 0.006 [136 – 600] *p* = 0.044

*All clusters were calculated from stimulus onset to 600 ms post-onset. Significant clusters indicated by their time windows (in brackets), with corresponding p-values. The dash indicates that no positive or negative clusters were observed in the data*.

Based on our hypotheses, we specified two 20ms time-windows centered around the peaks of the group average N1 and P2 components. We then used cluster analyses to determine over which electrodes between-session activity differed significantly between adapters and controls. Such electrodes were deemed likely to encode variation corresponding to the behavioral effects of interest. Testing this interaction was accomplished by first subtracting averaged ERPs in the post-training session from those in the pre-training session for each group and stimulus step (Figures 4D,E, top and middle panels). We then performed cluster analyses comparing the difference in activity in the two time-windows between groups (Figures 4D,E, top and middle panels). For stimulus one, significant clusters were found in the P2 window (*p* = 0.005, Figure 4D, bottom panel), but not the N1 window. Conversely, for stimulus three, significant clusters were found for the N1 window (*p* = 0.016, Figure 4E, bottom panel), but not the P2 window. For stimulus five, small clusters of activity were found for both N1 (electrodes: “Fp1”, “F7”, “Fp2”, “F3”, “Fz”; *p* = 0.029) and P2 (electrodes: “F4”, “T8”, “C4”, “P8”; *p* = 0.029).

Having identified candidate electrodes and time windows in which adapters and controls differed with regard to between-session variation in cortical amplitude, we then tested whether changes in the amplitude of these two components (averaged over all samples in the 20ms time window and all electrodes found to be active in the cluster for that stimulus) correlated with changes in standardized F1 for these three stimuli, with Bonferroni-corrections for the four tests.

**Figure 5:**
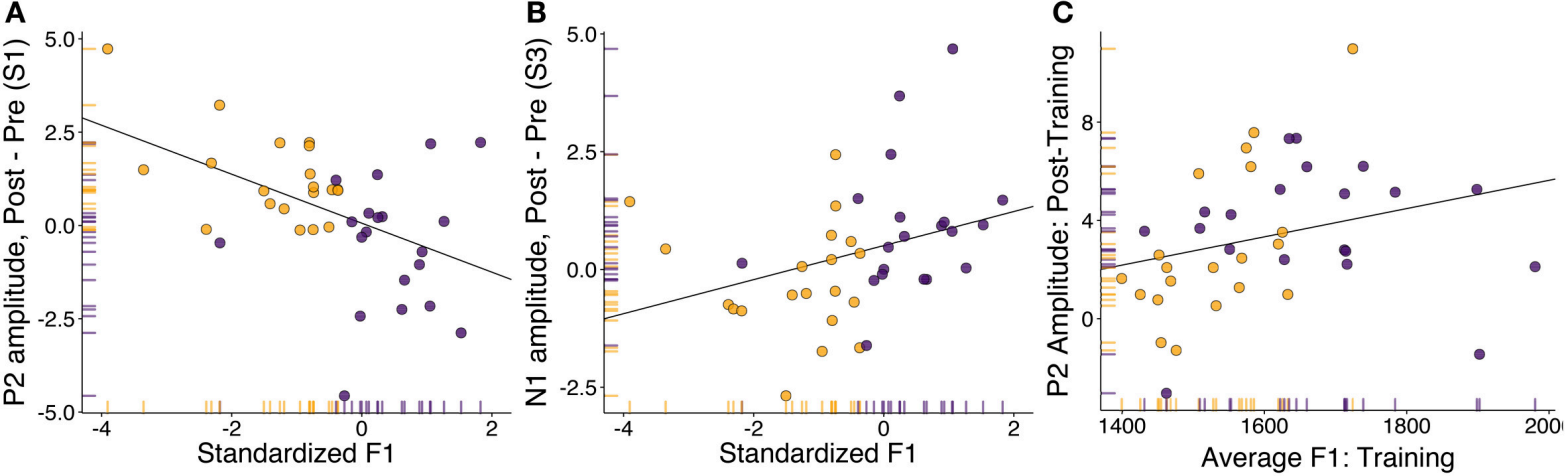
**Correlation analyses between speech motor behavior and neural component amplitude**. Adapters are shown in yellow, controls in purple. **(A)** Correlation between standardized F1 (representing change in F1 in the speech training session compared to baseline) and the difference in P2 amplitude during perception of the trained vowel (step one). **(B)** Correlation between standardized F1 (representing change in F1 in the speech training session compared to baseline) and the difference in N1 (1ms) amplitude during perception of the most ambiguous vowel (step three). **(C)** Correlation between average F1 in the speech training task and P2 amplitude averaged over all stimuli in the post-training identification session.

For stimulus step one (clear [ɛ]), we found a significant correlation between changes in P2 amplitude and standardized F1 (Figure 5A: *rho* = −0.43, *p* = 0.006). Follow-up testing revealed no significant within-group correlations. The direction of the correlation indicated that participants who produced vowels with a lower F1 during the training session tended to show increases in P2 amplitude following training, and vice versa. This result runs counter to that found by Ito et al. (2016), in which compensatory adaptation led to *decreases* in P2 amplitude. However, it is important to note that the feedback shift in their study was opposite in direction to that utilized in this study. For stimulus step three (most ambiguous step), changes in N1 amplitude were also found to correlate with standardized F1 (Figure 5B: *rho* = 0.44, *p* = 0.005). These correlations were not significant within either group alone. As most of the between-session changes in perception were localized to this stimulus step (Figure 2B), this might indicate that changes in amplitude of this earlier component drove changes in perception. For stimulus step five (clear [ɪ]), standardized F1 was not found to correlate with either N1 amplitude (*r* = 0.018, *p* = 0.91) or P2 amplitude (*r* = −0.009, *p* = 0.96). Overall, these correlations indicate that changes in cortical responses to specific stimulus steps (one and three) were related to changes in vowel production.

Having also found some evidence that overall proportion of [ɪ] responses in the baseline session may have been related to produced F2 (Figure 3A), we explored whether F1 and F2 values in the baseline and training session were related to cortical amplitude averaged over all stimulus steps. Utilizing the same electrodes found to be significant for N1 (stimulus three) and P2 (stimulus one), we averaged over 20ms time-windows corresponding to peak of the grand average N1 (104–124 ms) and P2 (192-212ms) components. Our exploratory tests examined relationships between average component amplitude (N1, P2) and formant production (F1, F2) in both sessions (pre, post), totaling eight comparisons. Partial correlations were utilized to control for the correlated nature of the formants, and Holm-Bonferroni corrections were applied to control for multiple comparisons.

No significant relationships were found, in either session, between average N1 amplitude and formant production. A weak correlation was found between average produced F1 in the training session and average P2 amplitude in the post-training categorization task. The correlation approached significance (Figure 5C; *rho* = 0.41, *p* = 0.01), and became significant after three multivariate outliers were removed (*r* = 0.47, *p* = 0.003, *BF*_10_ = 12.9). While caution needs to be taken with regard to the removal of outliers, this correlation suggests that the F1 frequency of the vowel produced during training was related to average P2 amplitude in the post-training perceptual task.

To summarize the results of the neurobehavioral analyses, we found no evidence of within-session, between-group differences in cortical amplitude. However, by-stimulus cluster-analyses suggests that, for specific stimuli, between-session changes in cortical responses varied between adapters and controls. Targeted analyses revealed that for stimulus one, the clear [ɛ] stimulus that was also the training vowel, standardized F1 correlated with changes in P2 amplitude. Yet for stimulus three, the most ambiguous stimulus, changes in standardized F1 were found to correlate with changes in N1 amplitude. These correlations were not significant within groups, suggesting that they related to overall changes in produced F1 rather than exposure to altered auditory feedback. While the behavioral data suggested a possible relationship in between F2 and identification responses, neither N1 or P2 amplitude was found to correlate with produced F2 in either session. When averaging over all stimulus steps, the only relationship between behavioral and electrophysiological measures for which there was any evidence was between average F1 in the training task and average P2 amplitude in the post-training session.

## Discussion

This study investigated to what extent phonetic categorization reflects one's sensorimotor experience. Primarily, we examined whether perceptual shifts associated with speech production training reflect changes in earlier cortical response components associated with acoustic feature extraction (Tavabi et al., 2007; Obleser and Eisner, 2009; Chang et al., 2010), or later processes associated with phonetic categorization (Bidelman et al., 2013) and perceptual learning (Tremblay et al., 2014). We tested whether altering how a vowel is produced modulates perceptual and electrophysiological responses during phonetic categorization. While between-group differences in phonetic categorization failed to reach significance, we found some evidence linking behavior in the production tasks to behavior in the phonetic categorization tasks. In addition to these relationships between production and perception, we also found neurobehavioral correlations between phonetic variables and the amplitude of both early and late ERP components.

Primarily, we found that between-session changes in production correlated with changes in both early (N1) and late (P2) auditory components, though only for specific stimulus steps. We also found some evidence hinting at a relationship between average F1 production in the training session and P2 amplitude in the subsequent identification task. Thus, the results of the exploratory analyses indicate that sensorimotor experience may affect early vowel decoding processes (Obleser and Eisner, 2009) as well as later perceptual decision processes (Bidelman et al., 2013; Mostert et al., 2015). We suggest that the observed pattern of results can be best characterized as a remapping of the relationship between acoustics and articulation by sensorimotor experience. Before arguing for this interpretation, we first consider possible reasons why the group-level results differed from those found previously.

In contrast to the findings reported in Lametti et al. (2014), our results did not reveal any significant between-group differences in phonetic categorization. Specifically, adapters did not differ significantly from controls with respect to changes in phonetic categorization following speech production training. Furthermore, though the correlation analyses suggested a possible relationship between changes in F1 and changes in phonetic categorization, the direction of the correlation conflicts with that found in Lametti et al. (2014). However, given that our correlations did not reach significance after applying Holm-Bonferroni corrections, and that they only held when both adapters and controls were included, it is difficult to draw conclusions about the differences between these two studies.

The results of our exploratory analyses suggested that, within each session, F2 was a stronger predictor of overall phonetic categorization than F1 (Figure 3). This may have been due to the fact that the experiment involved Dutch rather than English speaking participants. In addition to a distinction between [ɛ] and [ɪ], Dutch also has a rounded vowel [ʏ], distinguished from [ɪ] by primarily F2 and F3 (Adank et al., 2004). The presence of this phonological contrast may have increased Dutch participants' attention to the value of F2 compared to English participants. It is possible that this attention to F2 may have counteracted any group-level differences induced by changes in F1.

Another reason for lack of between-group behavioral effects in our study may have come from differences in stimulus design. Lametti et al. (2014) utilized a ten-step phonetic continuum, whereas the present study utilized a five-step continuum based on Bidelman et al. (2013). While standardized F1 was found to correlate with changes in phonetic categorization, correlations between this formant and changes in the amplitude of the P2 component were only found for stimulus step one. As a continuum endpoint, this stimulus exhibited almost no differences in behavioral responses before and after training. It may be that clearer between-group differences would have emerged had we utilized a ten-step continuum, increasing the number of stimuli closer to [ɛ].

Finally, both sensorimotor adaptation (Rochet-Capellan and Ostry, 2011; Rochet-Capellan et al., 2012) and phonetic recalibration (Eisner and McQueen, 2005; Reinisch et al., 2014) have been found to be extremely specific. Given that we used a full word (“pet”) during the speaking task, yet presented participants with isolated vowels (as in Bidelman et al., 2013), the lack of group-level effects may be due to differences between the training and test stimuli. The effects may have been stronger had we used a “pet-pit” continuum for the perceptual task, or modulated the acoustics of the stimuli to match the gender of the participant.

Returning to the results of the exploratory analyses, we found that changes in the amplitude of the N1 and P2 components during phonetic categorization were related to changes in formant production between the baseline and training tasks. For the N1 component, this relationship only held for stimulus step three (the most ambiguous stimulus), while for the P2 component, this relationship only held for stimulus step one (clear [ɛ]). Though the correlations suggested that changes in component amplitude were indeed driven by behavior in the preceding speech production task, it is important to consider whether the results might also be explained by other factors.

One possibility is that the effects reflect exposure to the categorization stimuli. In previous auditory training experiments, simple exposure to a phonetic continuum has been found to elicit increases in P2 amplitude (Tremblay et al., 2010, 2014). This pattern has been associated with the formation of a new phonetic contrast. However, the current study utilized an existing phonetic contrast, and furthermore, found diverging results for participants based on their behavior during the speech production task. When listening to the same [ɛ]-vowel after speech production training, P2 amplitude increased for participants who had produced this vowel with a lower F1 during training, yet decreased for participants who had produced this vowel with a higher F1. Therefore, it is unlikely that the observed effects can be simply attributed to exposure to the phonetic continuum.

Another possibility is that the effects reflect selective adaptation and/or phonetic recalibration in response to the categorization stimuli. Adapters received altered auditory feedback, leading to a mismatch between production and perception as well as a change in auditory feedback, while controls received unaltered feedback. Therefore, it could be argued that changes in amplitude observed in the control participants may reflect selective adaptation, having repeated the same vowel multiple times, while changes in amplitude observed in adapters may reflect phonetic recalibration of the trained vowel (van Linden et al., 2007; Shiller et al., 2009; Kleinschmidt and Jaeger, 2016). Repeated presentation of the same stimulus (or stimulus type) elicits ERP components with diminished amplitude compared to a novel stimulus (Belin and Zatorre, 2003; Tian and Poeppel, 2010, 2013). While this has previously been ascribed to “fatiguing” of feature detectors (Eimas and Corbit, 1973; Samuel, 1986), recent modeling suggests that speech sound categories may be likened to probability density functions, which are updated on the basis of the distribution of input exemplars (Kleinschmidt and Jaeger, 2015). Therefore, in controls, repeated production of unaltered [ɛ] may have sharpened feature representation for this vowel, leading to less activity when these features are matched (stimulus one) and increased activity for more ambiguous stimulus steps (e.g., step three, Figure 4D). Conversely, in adapters, the error between expected and heard feedback may have led to a shift in the center of this distribution, leading to increased error during perception. If this were true, then we would expect to observe increases in the amplitude of all components as a consequence of adaptation to altered feedback, and a decrease in the amplitude of these components as a consequence of unaltered feedback. Accordingly, most adapters exhibited increases in P2 amplitude in response to stimulus one. Yet, while most adapters exhibited *decreases* in N1 amplitude in response to stimulus three, many controls exhibited *increases* in the amplitude of this component (Figure 5B). Furthermore, in a similar experiment, Ito et al. (2016) found decreases in P2 amplitude after adaptation to altered feedback. The opposing effects observed in these results cannot be accounted for by selective adaptation and phonetic recalibration alone.

We therefore propose that changes in component amplitude in both groups reflected changes in the mapping between acoustic values and articulatory features (Poeppel et al., 2008; Tourville et al., 2013). For example, the amplitude of the P2 component has been found to be greater when an ambiguous stimulus was categorized as a low vowel [a] than when this same acoustic stimulus is categorized as a high vowel [u] (Bidelman et al., 2013). As stated in the introduction, if the vowel [ɛ] in “pet” comes to be associated with a more closed or open jaw position due to changes in production (regardless of feedback), then a sensory-to-motor mapping account would predict that P2 amplitude should increase or decrease correspondingly.

In Ito et al. (2016), the value of F1 was decreased for participants in the altered feedback condition, leading adapters to produce the target [ɛ]-vowel with a greater F1 frequency compared to baseline (i.e., as a more [æ]-like vowel, in which the jaw is lower). Greater adaptation responses corresponded to greater *decreases* in the amplitude of the P2 component when listening to the trained vowel. In the present experiment, the experimental manipulation consisted of an increase in the value of F1, leading adapters to produce a more [ɪ]-like vowel. Greater adaptation responses were found to correspond to greater *increases* in P2 amplitude. These correlations suggest that the direction of change in the amplitude of the P2 component during perception of the trained vowel corresponded to those expected when perceiving a vowel with a specific height (Shestakova et al., 2004; Bidelman et al., 2013).

Based on the results of Bidelman et al. (2013), we can re-characterize the observed changes in P2 amplitude for clear [ɛ] in Ito et al. (2016) and the current experiment. Articulating [ɛ] as more closed, [ɪ]-like vowel during production (as in the present experiment) caused a previously presented auditory stimulus to be perceived as if it were a more open vowel. Articulating [ɛ] as a more open, [æ]-like vowel led the test stimulus to be perceived as if it were a more closed vowel (Ito et al., 2016).

One might object that rather than attributing these changes to the articulatory behavior, they can be attributed to the auditory feedback participants experienced during training. That is, rather than having a sensorimotoric cause, the results could simply reflect exposure to auditory feedback during production. But a purely sensory explanation is unable to account for the modulation of the N1 and P2 components observed in adapters. Speakers have been found, in response to similar feedback shifts, to only partially compensate for shifts in auditory feedback (Katseff et al., 2012). In the present study, adaptation opposing the direction of the shifted feedback compensated on average for 38% shift in auditory feedback. This means that despite producing a vowel with a lower F1, participants nevertheless heard themselves producing a vowel with a higher F1 value than normal (i.e., similar to what the control group produced and heard). In contrast to the observed pattern of results, a purely sensory account would predict that the direction of the effect should be the same for both groups or that effects should be strongest for participants who adapted less, neither of which were found to be the case. That being said, the variation observed in amplitude of these components was not completely accounted for by the changes in production. Speakers have been found to differ with regard to their dependence on somatosensory compared to auditory feedback in a production task (Lametti et al., 2012). It may be the case that individual differences in sensory preference modulated the observed correlation.

Throughout this paper, we have consistently referred to our perceptual task as phonetic categorization rather than speech perception. This choice reflects the fact that effects observed in phonetic categorization tasks do not necessarily coincide with those observed in other, possibly more natural speech contexts (Hickok and Poeppel, 2000; Norris et al., 2000; Hickok et al., 2009; Krieger-Redwood et al., 2013). Therefore, in order to generalize our results beyond phonetic categorization, it is important to consider how this task may resemble natural speech perception. The current experiment employed a two-alternative forced choice task, leading to a categorical response profile with a rather sharp identification boundary (Liberman et al., 1957; Chang et al., 2010; Goldstone and Hendrickson, 2010; Bidelman et al., 2013). The experimental design therefore led participants to focus on acoustic cues relevant for distinguishing the target contrast. However, the ‘categoricalness’ of categorical perception may diminish or even disappear when more response options are available (Lotto, 2000; Schouten et al., 2003; Gerrits and Schouten, 2004). This suggests that mapping acoustics onto articulatory representations based on one's own sensorimotor experience may not apply to situations where the range of possible categories to which an acoustic signal can be assigned is more open, as in natural speech. In such cases, it may be better to rely on lexical information to reinterpret acoustics (e.g., Ganong, 1980; Norris et al., 2003). Yet when context constrains the range of possible sound categories, simulation of candidate phonetic categories may aid perception (Poeppel et al., 2008; Tian and Poeppel, 2013).

We have argued that the cortical and perceptual effects observed in this study reflect a mapping of acoustics onto articulation in order to classify a speech sound, and that these mappings may be updated by recent sensorimotor experience. Therefore, in addition to supporting and maintaining speech production abilities (Lane and Webster, 1991; Niziolek et al., 2013), sensorimotor experience may play a role in certain perceptual contexts as well. As has been noted, “the task of perceiving speech sounds is complex and the ease with which humans acquire, produce and perceive these sounds is remarkable” (Carbonell and Lotto, 2014). It is equally remarkable that humans are able to swiftly and flexibly take advantage of diverse cues and resources in order to deal with the perceptual task at hand (Erb et al., 2013; Brown and Kuperberg, 2015). Listeners have been argued to draw on their knowledge about how speech is produced in order to help decode speech under difficult listening conditions (Nuttall et al., 2016) and as a tool to predict how upcoming speech will sound (Brunellière et al., 2009; Tian and Poeppel, 2010, 2013). What this exploratory study has suggested is that this knowledge is not static, but is updated and modulated by our ongoing sensorimotor experiences.

## Ethics statement

This study was carried out in accordance with the recommendations of the Ethics Committee of the Social Sciences Faculty of Radboud University. All participants gave written informed consent in accordance with the Declaration of Helsinki. Participations were informed that their participation was voluntary and that they were free to withdraw from the study at any time without any negative repercussions and without needing to specify any reason for withdrawal. All were reimbursed for their participation.

## Author contributions

WS designed and performed the experiment and analyzed the data. WS, JM, and AM interpreted the results. WS wrote the manuscript. JM and AM performed critical revisions, and approved manuscript for publication.

## Funding

Funding for this research was provided by the Max-Planck-Gesellschaft.

### Conflict of interest statement

The authors declare that the research was conducted in the absence of any commercial or financial relationships that could be construed as a potential conflict of interest.
